# Lymphadenitis caused by infection with an isoniazid- and rifampin-resistant strain of *Mycobacterium bovis *BCG in an infant with IFN-γ/IL-12 pathway defect*

**DOI:** 10.1590/S1806-37132014000200014

**Published:** 2014

**Authors:** Lilian Martins Oliveira Diniz, Tiago Guimarães, Maria das Graças Rodrigues de Oliveira, Jorge Andrade Pinto, Silvana Spindola de Miranda

**Affiliations:** Federal University of Minas Gerais School of Medicine, Belo Horizonte, Brazil; Federal University of Minas Gerais School of Medicine Hospital das Clínicas, Belo Horizonte, Brazil; Federal University of Minas Gerais School of Medicine Hospital das Clínicas, Belo Horizonte, Brazil; Federal University of Minas Gerais School of Medicine, Belo Horizonte, Brazil; Federal University of Minas Gerais School of Medicine, Belo Horizonte, Brazil

**Keywords:** BCG vaccine, Interferon-gamma, Tuberculosis, multidrug-resistant

## Abstract

We report a rare case in a female infant (age, 3.5 months) with primary immunodeficiency (IFN-γ/IL-12 pathway defect) who presented with suppurative lymphadenitis after *Mycobacterium bovis *BCG vaccination. The strain of *M. bovis* BCG identified was found to be resistant to isoniazid and rifampin. The patient was treated with a special pharmacological regimen involving isoniazid (in a limited, strategic manner), ethambutol, streptomycin, and IFN-γ, after which there was complete resolution of the lesions.

## Introduction

BCG is an attenuated strain of *Mycobacterium bovis* that is present in the tuberculosis vaccine, which was first used in humans in 1922.^(^ ^1^ ^)^ The vaccine produces an artificial primary infection with non-virulent bacilli in order to increase resistance to a future infection with virulent bacilli.^(^ ^1^ ^)^

The World Health Organization recommends vaccination with the BCG vaccine for all newborns in areas with a high prevalence of tuberculosis as a means to prevent the disease.^(^ ^2^ ^)^ In Brazil, the use of the BCG vaccine for many years has demonstrated the effectiveness of vaccination, with minimal adverse reactions, and with severe complications occurring only rarely.^(^ ^1^ ^)^

During the natural course of the vaccination lesion, nonsuppurative axillary, supra-axillary, or infraclavicular lymph node swelling can be seen. However, severer lesions caused by *M. bovis* BCG strains can be found in patients with immunodeficiency, who should be treated with a combination regimen of drugs, such as isoniazid, rifampin, ethambutol, and ciprofloxacin.^(^ ^1^ ^,^ ^3^ ^-^ ^5^ ^)^

The treatment of disease caused by BCG can be complicated by resistance to pyrazinamide, which is inherent to all strains of *M. bovis*, as well as by intermediate resistance of some strains to isoniazid and by the emergence of acquired resistance during inappropriate therapy.^(^ ^5^ ^,^ ^6^ ^)^

The authors report a case of an infant with primary immunodeficiency who had suppurative lymphadenitis after *M. bovis* BCG vaccination. The strain of *M. bovis* BCG identified was found to be resistant to isoniazid and rifampin.

## Case report

**Figure 1:**
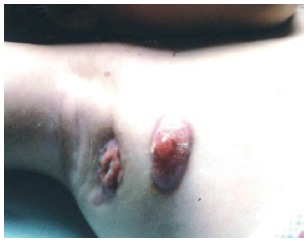
Suppurative right axillary and infra-axillary lesions after BCG vaccination.

A female infant (age, 3.5 months) was brought by her mother to the Department of Pediatric Infectious Diseases of the Federal University of Minas Gerais *Hospital das Clínicas* because of "inflammation" at the BCG vaccination site. It was reported that there was a family history of two cousins who had experienced the same adverse event after BCG vaccination and had died with suspected primary immunodeficiency in the first year of life. The physician who treated the infant noted the presence of a granulomatous lesion (not suggestive of secondary infection) at the vaccination site, as well as ipsilateral suppurative lymphadenitis (Figure 1), and the patient was started on isoniazid therapy (10 mg/kg daily for 45 days). The lymphadenitis resolved during treatment. However, after discontinuation of the drug, the lesion reappeared. The patient was prescribed isoniazid for two additional months. The lesion resolved, but, at the end of drug treatment, it returned.

The infant was referred to the Immunodeficiency Outpatient Clinic of the Federal University of Minas Gerais *Hospital das Clínicas* with suspected primary immunodeficiency. Initial immunological assessment showed that serum immunoglobulin levels and lymphocyte subpopulation levels were within the normal range. Because the initial immunological profile was normal and the clinical course was relatively benign, we hypothesized that the patient had an IFN-γ/IL-12 pathway defect. Serology for HIV was negative.

At that same time, we chose to biopsy the affected lymph node. Smear microscopy and mycobacterial culture of these clinical specimens were requested. Because of the isoniazid-induced reactivation of the lesion and the family history of primary immunodeficiency, the patient was started on a combination regimen of isoniazid (10 mg/kg daily), rifampin (10 mg/kg daily), and ethambutol (25 mg/kg daily). Ethambutol was introduced after a literature review, which showed that this drug is introduced in severer forms of infection with *M. bovis* BCG.^(^ ^1^ ^,^ ^3^ ^-^ ^5^ ^)^ The infant was also referred to the department of ophthalmology for evaluation because of the risk of ethambutol-induced optic neuritis. In the second month of treatment, there was optic disc blurring bilaterally. At that same time, the results of smear microscopy and culture of the lymph node biopsy specimen were positive for the *M. tuberculosis* complex, and susceptibility testing showed resistance to isoniazid. In view of the partial resolution of lymphadenitis and the possibility of drug-induced optic neuritis, ethambutol was discontinued, and rifampin and isoniazid were continued despite resistance to isoniazid.

In the eighth month of treatment, the infant was hospitalized with secondary infection at the biopsy site. A chest CT performed during hospitalization showed diffuse coalescent axillary lymphadenopathy on the right, with necrotic and fistulized lymph nodes, which indicated lesion activity. A second lymph node biopsy was performed, and the specimen was sent to the Professor Hélio Fraga Referral Center, located in the city of Rio de Janeiro, Brazil, for mycobacterial and molecular analysis of the strain. The laboratory test results showed that it was the *M. bovis* BCG strain. The identification tests used were basic biochemical tests and polymerase chain reaction restriction analysis of the *hsp65* gene. Susceptibility testing was performed in BACTEC Mycobacteria Growth Indicator Tube (MGIT) 960 (Becton Dickinson, Sparks, MD, USA), the result of which showed resistance to isoniazid and also to rifampin. Sequencing of the *rpo*B gene detected the D516V mutation, and sequencing of the *kat*G gene detected the S315T mutation.

Given the results, we chose to continue treatment with isoniazid, restart ethambutol, and have the patient have ophthalmic follow-up weekly, as well as to add 30 doses of streptomycin (25 mg/kg daily) on alternate days for six months and discontinue rifampin. The ophthalmic lesion remained stable throughout the treatment period. Given the difficulty of the case, streptomycin was used because it is bactericidal and it is a first-line drug, as well as because there is a lack of knowledge about the true *in vivo* response to isoniazid.

The hypothesis of IFN-γ/IL-12 pathway defect was confirmed by molecular tests that detected a homozygous mutation in IL-12 receptor β_1_, thus excluding probable severe combined immunodeficiency (SCID). Therefore, subcutaneous IFN-γ three times weekly was added to the treatment regimen.

**Figure 2:**
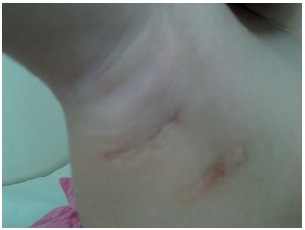
Healed lesions in the right axillary and infra-axillary regions after treatment.

After six months of treatment, the axillary lesion had completely resolved (Figure 2). At this writing, three years after the onset of the condition, the patient remained free of lymphadenitis, and the ocular lesion remained stable, with no impact on vision.

## Discussion

Adverse reactions to the BCG vaccine vary according to the type of strain, bacterial load, administration of the vaccine, and host characteristics. Nonsuppurative reactive lymphadenitis can occur in children in the first months after administration of the vaccine and is mostly due to incorrect technique of administration. In Brazil, isoniazid has been used in the treatment of suppurative lymphadenopathy secondary to the vaccine in immunocompetent patients. This recommendation is based on the fact that the Brazilian strain of BCG is usually sensitive to this drug in in vitro tests.^(^ ^7^ ^)^ However, in the present case, the BCG strain proved to be resistant to isoniazid and rifampin, showing probable *in vivo* response to the combination of these drugs with other drugs.

Nevertheless, the vaccine can cause complications that are more severe and, in most cases, occur in patients with immunosuppression. The incidence of disseminated *M. bovis* BCG disease in European countries is estimated to be two cases per million children vaccinated, and the disease occurs only occasionally in immunocompetent children.^(^ ^8^ ^)^ In their study, Talbot et al. reported a rate of immune defects of 86% in children diagnosed with disseminated disease.^(^ ^9^ ^)^ In the literature, there have been reports of complications in HIV-infected patients and in patients with primary immunodeficiency.^(^ ^4^ ^,^ ^7^ ^)^ Hesseling et al. described a series of 25 patients diagnosed with severe *M. bovis* BCG infection associated with primary immunodeficiency.^(^ ^10^ ^)^ Santos et al. described the cases of three patients with vaccine-induced infection, one of whom had IFN-γ/IL-12 pathway deficiency.^(^ ^3^ ^)^ An IFN-γ/IL-12 pathway defect is an immunodeficiency disorder in which there is increased susceptibility to infections with microorganisms of the genera *Mycobacterium* and *Salmonella*.^(^ ^11^ ^,^ ^12^ ^)^ It is classified as a congenital defect of phagocyte number, function, or both. Host defenses against these bacteria strongly depend on the functional integrity of mononuclear phagocytes and their interaction with T lymphocytes. T lymphocytes and natural killer cells of affected patients express a defective IL-12 receptor on their cell surfaces, leading to low production of IFN-γ, which is the major factor responsible for mycobacterial death.^(^ ^13^ ^)^ The diagnosis of IFN-γ/IL-12 pathway defects requires a tiered approach and laboratory support.^(^ ^14^ ^)^ In patients with severe disseminated mycobacterial infection, other immunodeficiency disorders, such as severe combined immunodeficiency, should be excluded first. In some cases, the treatment of IFN-γ/IL-12 pathway defects requires aggressive use of drugs against the mycobacterium and subcutaneous IFN-γ replacement therapy as a treatment option.^(^ ^11^ ^)^ In the present case report, ancillary tests confirmed this deficiency, which favored the development of lesions due to *M. bovis* BCG.

A system for classification of *M. bovis* BCG disease in immunocompromised patients was developed by Talbot et al. and subsequently revised by Hesseling et al.^(^ ^9^ ^,^ ^10^ ^)^ Infection is classified on the basis of its presentation as local, regional, distant, or disseminated disease. Regional disease is defined as that in which there is a lesion at the vaccination site and ipsilateral regional lymph node involvement, with lymphadenopathy and fistula formation and/or suppuration, as in the case described here.^(^ ^9^ ^,^ ^10^ ^)^

We report the course of *M. bovis* BCG infection in an infant with primary immunodeficiency who presented with a regional lesion located ipsilateral to the vaccination site. The lesion was unresponsive to the recommended treatment with isoniazid, and the strain identified was found to be resistant to two drugs (rifampin and isoniazid). Although susceptibility testing showed resistance to isoniazid, we chose to continue treatment with this drug, since testing using a critical concentration does not quantify the level of resistance (low, moderate, or high), which is determined using a minimum inhibitory concentration (MIC).^(^ ^15^ ^)^ The level of *in vitro* resistance to isoniazid was not quantified, since an MIC was not performed, as well as not reflecting the *in vivo* reality, and the mutation found (the *kat*G S315T mutation) may be related to moderate resistance to isoniazid. Since ethambutol was withdrawn from the regimen because of suspected optic neuritis, there may have been strain selection, with acquisition of resistance to rifampin. Rifampin was discontinued, given that the mutation found in the *rpo*B region (the D516V mutation) is in most cases related to a high level of resistance.^(^ ^15^ ^)^ There have been studies of mutations and MIC associated with clinical response for *M. tuberculosis*, but there have been no reports regarding *M. bovis* BCG.

Ethambutol-induced optic neuritis was ruled out, since the alteration in the optic disk remained stable throughout the follow-up period. The favorable outcome was possible, despite resistance to isoniazid, because of the combination of streptomycin and ethambutol, as well as because of the inclusion of the immunomodulator, resulting in resolution of the lesions.
