# Supplementary material for: Lymphadenitis caused by infection with an isoniazid- and rifampin-resistant strain of Mycobacterium bovis BCG in an infant with IFN-γ/IL-12 pathway defect
Source: J Bras Pneumol. 2014 Mar-Apr;40(2):188–92. doi: 10.1590/S1806-37132014000200014 (PMC4083635; doi:10.1590/S1806-37132014000200014)
Supplement: Translation

# Translation: Linfadenite por *Mycobacterium bovis *BCG resistente a isoniazida e rifampicina em lactente com defeito no eixo IFN-γ/IL-12*

**Authors:** Lilian Martins Oliveira Diniz, Tiago Guimarães, Maria das Graças Rodrigues de Oliveira, Jorge Andrade Pinto, Silvana Spindola de Miranda

**Affiliations:** Faculdade de Medicina, Universidade Federal de Minas Gerais, Belo Horizonte (MG) Brasil; Hospital das Clínicas, Faculdade de Medicina, Universidade Federal de Minas Gerais, Belo Horizonte (MG) Brasil; Faculdade de Medicina, Universidade Federal de Minas Gerais, Belo Horizonte (MG) Brasil; Faculdade de Medicina, Universidade Federal de Minas Gerais, Belo Horizonte (MG) Brasil; Faculdade de Medicina, Universidade Federal de Minas Gerais, Belo Horizonte (MG) Brasil

**Keywords:** Vacina BCG, Interferon gama, Tuberculose resistente a múltiplos medicamentos

## Abstract

Relatamos um caso raro em uma lactente com três meses e meio de idade, portadora de imunodeficiência primária (defeito no eixo IFN-γ/IL-12), que apresentou linfadenite supurativa após a vacinação por *Mycobacterium bovis *BCG, cepa essa resistente a isoniazida e rifampicina. Após o tratamento com um esquema medicamentoso especial com isoniazida (de forma estratégica e limitada), etambutol, estreptomicina e IFN-γ, houve a cura completa das lesões.

## Introdução

O BCG é uma cepa do *Mycobacterium bovis *atenuada presente na vacina contra a tuberculose, que foi inoculada em humanos pela primeira vez em 1922.^(^ ^1^ ^)^ A vacina provoca uma primoinfecção artificial, ocasionada por bacilos não virulentos, visando aumentar a resistência do indivíduo em face de uma infecção ulterior, causada por bacilos virulentos.^(^ ^1^ ^)^

A Organização Mundial da Saúde recomenda a vacinação com a vacina BCG em todos os neonatos de áreas com alta prevalência de tuberculose como forma de prevenção da doença.^(^ ^2^ ^)^ No Brasil, seu uso durante muitos anos demonstrou a eficiência da vacinação, com o mínimo de reações indesejáveis, só raramente induzindo o surgimento de complicações graves.^(^ ^1^ ^)^

Durante a evolução normal da lesão vacinal, o enfartamento ganglionar axilar, supraclavicular ou infraclavicular não supurado pode ser observado. No entanto, lesões mais severas causadas pelo *M. bovis* vacinal podem ser encontradas em pacientes com imunodeficiência, que devem ser tratados com um esquema de associação de fármacos, tais como isoniazida, rifampicina, etambutol e ciprofloxacina.^(^ ^1^ ^,^ ^3^ ^-^ ^5^ ^)^

O tratamento da doença causada pelo BCG pode ser complicado pela resistência à pirazinamida, que é inerente a todas as cepas de *M. bovis*, assim como pela resistência intermediária à isoniazida de algumas cepas e pela emergência de resistência adquirida durante terapias inapropriadas.^(^ ^5^ ^,^ ^6^ ^)^

Os autores relatam um caso de uma lactente portadora de imunodeficiência primária com linfadenite supurativa após vacinação pelo BCG, sendo essa cepa resistente a isoniazida e rifampicina.

## Relato de caso

Uma lactente de três meses e meio de idade veio trazida pela mãe ao Serviço de Infectologia Pediátrica do Hospital das Clínicas da Universidade Federal de Minas Gerais devido a uma "inflamação" no local da vacinação com BCG. Houve o relato de história familiar de dois primos que haviam apresentado o mesmo evento adverso após a vacinação com BCG e que vieram a falecer no primeiro ano de vida com suspeita de imunodeficiência primária. O médico que atendeu a lactente observou a presença de uma lesão granulomatosa (não sugestiva de infecção secundária) no local da vacina, além de linfadenite supurada ipsilateral (Figura 1), sendo iniciada então a terapia com isoniazida (10 mg/kg ao dia) por 45 dias. Houve resolução da linfadenite durante o tratamento; porém, após a suspensão da medicação, a lesão reapareceu. A isoniazida foi prescrita novamente por mais dois meses. A lesão regrediu mas retornou mais uma vez ao fim do uso da medicação.

**Figura 1:** Lesão supurativa axilar e infra-axilar direita após a vacinação com BCG.

A lactente foi encaminhada ao Ambulatório de Imunodeficiência do Hospital das Clínicas da Universidade Federal de Minas Gerais com suspeita de imunodeficiência primária. Foi submetida à avaliação imunológica inicial, demonstrando valores dentro da normalidade para a dosagem sérica de imunoglobulinas e de subpopulação de linfócitos. Em função da normalidade do perfil imunológico inicial e do quadro clínico de evolução relativamente benigna, foi aventada a hipótese de defeito no eixo IFN-γ/IL-12. A sorologia para o HIV foi negativa.

Na mesma época, optou-se pela realização de biópsia do linfonodo acometido. Foram solicitadas baciloscopia e cultura para micobactérias dessas amostras clínicas. Devido a reativação da lesão com o uso de isoniazida e a história familiar de imunodeficiência primária, foi iniciado um esquema de associação dos medicamentos isoniazida (10 mg/kg ao dia), rifampicina (10 mg/kg ao dia) e etambutol (25 mg/kg ao dia). O etambutol foi introduzido após uma revisão da literatura, a qual indicou que esse fármaco é introduzido em formas mais graves da infecção por *M. bovis* BCG.^(^ ^1^ ^,^ ^3^ ^-^ ^5^ ^)^ A lactente foi também encaminhada ao serviço de oftalmologia para avaliação devido ao risco de neurite óptica por etambutol. No segundo mês de tratamento, foi evidenciado borramento bilateral do disco óptico. Nessa mesma época, o resultado da baciloscopia e da cultura da biópsia de linfonodo mostraram-se positivos para o complexo *M. tuberculosis*, com teste de sensibilidade demonstrando resistência à isoniazida. Tendo em vista a melhora parcial da linfadenite e a possibilidade de neurite óptica medicamentosa, o etambutol foi suspenso, sendo mantidos rifampicina e isoniazida apesar da resistência a isoniazida.

No oitavo mês de tratamento, a lactente foi internada com infecção secundária no sítio da biópsia. A TC de tórax realizada durante a internação mostrou linfadenomegalia axilar direita difusa e coalescente, com linfonodos necróticos e fistulizantes, o que indicava lesão em atividade. Uma nova biópsia linfonodal foi realizada e encaminhada para a análise micobacteriológica e molecular da cepa ao Centro de Referência Professor Hélio Fraga, localizado na cidade do Rio de Janeiro. Os exames laboratoriais revelaram que se tratava da cepa do *M. bovis* BCG. Os testes de identificação realizados foram testes bioquímicos básicos e o método molecular *polymerase chain reaction restriction analysis *do gene* hsp65*. O teste de sensibilidade foi realizado em meio líquido automatizado BACTEC *Mycobacteria Growth Indicator Tube* (MGIT) 960 (Becton Dickinson, Sparks, MD, EUA), o qual revelou resistência a isoniazida e também a rifampicina. Foi realizado o sequenciamento dos genes *rpo*B, com detecção de mutação em D516V, e *kat*G, com mutação em S315T.

Diante dos resultados, optou-se pela manutenção do tratamento com isoniazida, retorno do uso de etambutol e acompanhamento oftalmológico semanal, além da associação de 30 doses de estreptomicina (25 mg/kg ao dia) em dias alternados por seis meses e suspensão do uso de rifampicina. A lesão oftalmológica manteve-se estável durante todo o tratamento. Diante da dificuldade do caso, a estreptomicina foi indicada por ser bactericida e de primeira linha, assim pela falta de conhecimento da real resposta da isoniazida in vivo*.*

A hipótese de defeito no eixo IFN-γ/IL-12 foi confirmada por testes moleculares que identificaram mutação em homozigose no receptor β_1_ de IL-12, excluindo uma provável imunodeficiência combinada grave (IDCG). Dessa forma, IFN-γ subcutâneo foi associado ao tratamento três vezes na semana.

Após seis meses de tratamento, a lesão axilar havia regredido completamente (Figura 2). Até o momento, três anos após o início do quadro, a paciente mantinha-se sem a linfadenite, e a lesão ocular permanecia estável, sem repercussões visuais.

**Figura 2:** Lesão cicatrizada na região axilar e infra-axilar direita após o tratamento.

## Discussão

As reações adversas à vacina BCG variam com o tipo da cepa, carga bacilar, administração da vacina e características do hospedeiro. A linfadenite não supurativa reacional pode ocorrer em crianças nos primeiros meses após a aplicação da vacina e decorre, em sua maioria, da técnica incorreta na aplicação. A isoniazida vem sendo empregada no Brasil para o tratamento da linfadenopatia supurada secundária à vacina em pacientes imunocompetentes. Essa recomendação baseia-se no fato de que a cepa brasileira da vacina BCG normalmente é sensível a essa droga em testes in vitro.^(^ ^7^ ^)^ Porém, no presente caso, foi demonstrada a resistência do bacilo BCG a isoniazida e rifampicina, com provável resposta in vivo quando associadas a outros fármacos.

No entanto, a vacina pode causar complicações mais graves e que, na maioria das vezes, ocorrem em pacientes com imunodepressão. Estima-se que a incidência de doença disseminada pelo *M. bovis* BCG em países da Europa seja de dois casos para cada 1.000.000 de crianças vacinadas, ocorrendo apenas ocasionalmente em crianças imunocompetentes.^(^ ^8^ ^)^ Talbot et al. descreveram em seu estudo uma taxa de 86% de defeitos imunológicos em crianças com diagnóstico da doença disseminada.^(^ ^9^ ^)^ Existem casos relatados na literatura de complicações em pacientes infectados pelo HIV e em pacientes com imunodeficiência primária.^(^ ^4^ ^,^ ^7^ ^)^ Hesseling et al. descreveram uma série de 25 pacientes diagnosticados com infecção grave por *M. bovis* vacinal associada à imunodeficiência primária.^(^ ^10^ ^)^ Santos et al. descreveram três casos de pacientes com a infecção vacinal, sendo um deles com deficiência no eixo IFN-γ/IL-12.^(^ ^3^ ^)^ O defeito no eixo IFN-γ/IL-12 é uma imunodeficiência na qual há uma susceptibilidade aumentada a infecções por microrganismos dos gêneros *Mycobacterium* e *Salmonella*.^(^ ^11^ ^,^ ^12^ ^)^ É classificada como um defeito congênito de fagócitos em número, função ou ambos. As defesas do hospedeiro para essas bactérias dependem fortemente da integridade funcional dos fagócitos mononucleares e sua interação com os linfócitos T. Linfócitos T e células *natural killer* de pacientes afetados expressam um receptor de IL-12 defeituoso em suas superfícies celulares, levando a uma baixa produção de IFN-γ, sendo esse o principal fator responsável pela morte da micobactéria.^(^ ^13^ ^)^ O diagnóstico dos defeitos no eixo IFN-γ/IL-12 requer uma abordagem escalonada e com apoio laboratorial.^(^ ^14^ ^)^ Em pacientes com infecção micobacteriana disseminada e grave, deve-se primeiramente excluir outras imunodeficiências, como a imunodeficiência combinada grave. Em alguns casos, o tratamento de defeitos no eixo IFN-γ/IL-12 exige o uso agressivo de fármacos contra a micobactéria e a reposição com IFN-γ subcutâneo como uma opção terapêutica.^(^ ^11^ ^)^ No relato do presente caso, demonstramos por exames complementares essa deficiência, o que favoreceu o desenvolvimento da lesão pelo *M. bovis* BCG.

Um sistema de classificação da doença causada pelo *M. bovis* vacinal em pacientes imunossuprimidos foi desenvolvido por Talbot et al. e posteriormente revisado por Hesseling et al.^(^ ^9^ ^,^ ^10^ ^)^ A infecção é classificada de acordo com sua apresentação em doença local, regional, distante e disseminada. A doença regional é descrita como aquela com lesão no local de inoculação da vacina e envolvimento de linfonodo regional ipsilateral, com linfadenomegalia, supuração e/ou formação de fístulas, conforme observado no caso descrito.^(^ ^9^ ^,^ ^10^ ^)^

Relatamos a evolução da infecção pelo *M. bovis* vacinal em uma lactente com imunodeficiência primária que apresentou lesão regional ipsilateral à vacina, não responsiva ao tratamento preconizado com isoniazida, sendo a cepa resistente a dois fármacos (rifampicina e isoniazida). Apesar de o resultado do teste de sensibilidade ter demonstrado resistência a isoniazida, optou-se pela manutenção dessa medicação já que testes utilizando uma concentração crítica não revelam a realidade quantitativa da resistência (baixa, moderada ou alta), a qual é determinada por *minimum inhibitory concentration* (MIC, concentração inibitória mínima).^(^ ^15^ ^)^ Não há como afirmar a relação quantitativa in vitro da resistência a isoniazida já que a MIC não foi realizada, e a mutação encontrada pode estar relacionada à resistência moderada a isoniazida (*kat*G, mutação S315T), além de não refletir a realidade in vivo. Com a retirada do etambutol devido a suspeita de neurite óptica, pode ter havido uma seleção das cepas, com aquisição da resistência a rifampicina. A rifampicina não foi mantida, pois a mutação encontrada na região *rpo*B (mutação D516V) está na grande maioria das vezes relacionada a um alto nível de resistência.^(15) ^Estudos de mutações e de MIC associados à resposta clínica foram descritos para o *M. tuberculosis*, mas não há relatos na literatura referentes ao *M. bovis* BCG.

Foi descartada a neurite óptica pela ação de etambutol, já que a alteração do disco óptico manteve-se estável durante todo o acompanhamento da lactente. O desfecho favorável, apesar da resistência a isoniazida, foi possível pela associação de estreptomicina e etambutol, assim como pela inclusão do imunomodulador, o que possibilitou a cura das lesões.
